# Supplementary material for: Staphylococcus epidermidis RP62A’s Metabolic Network: Validation and Intervention Strategies
Source: Metabolites. 2022 Aug 28;12(9):808. doi: 10.3390/metabo12090808 (PMC9503974; doi:10.3390/metabo12090808)
Supplement: Supplementary file 1 [file metabolites-12-00808-s001.zip › metabolites-1862050-supplementary.pdf]

# Supplementary Materials

## 1 Portability from ScrumPy to COBRApy

The model was obtained from the supplementary material provided by Díaz Calvo et al .Concretely, file GSM_SupIII.sbml.

**Adaptation of the model**. Originally the model was generated using ScrumPy (a well-known metabolic modeling package. However). To read this file into COBRApy some issues have to be solved:

- COBRApy did not recognize irreversible reactions of the model
- OBRApy generates automatically exchange reactions (with the prefix 'EX_' in their ids) to balance external metabolites declared in the model

To tackle them, some modifications on the SBML file need to be made

- Substitution of the SBML's version of the file (version 3.2 to version 3.1). The heading starts like this:

```
<?xml version="1.0" encoding="UTF-8"?>
<sbml xmlns="http://www.sbml.org/sbml/level3/version2/core"
level="3" version="2">
  <model>
</listOfParameters>
```

  The modification was as it follows:

```
<?xml version="1.0" encoding="UTF-8"?>
<sbml xmlns:fbc="http://www.sbml.org/sbml/level3/version1/fbc/
version2" xmlns="http://www.sbml.org/sbml/level3/version1/core"
level="3" version="1" sboTerm="SBO:0000624" fbc:required="false">
  <model fbc:strict="true" id="Staphylococcus epidermidis">
```

- Addition of a few lines to declare lower and upper bounds of the values that can take reactions fluxes (as COBRApy expects). Those parameters go right after the heading:

```
<?xml version="1.0" encoding="UTF-8"?>
<sbml xmlns:fbc="http://www.sbml.org/sbml/level3/version1/fbc/
version2" xmlns="http://www.sbml.org/sbml/level3/version1/core"
level="3" version="1" sboTerm="SBO:0000624" fbc:required="false">
  <model fbc:strict="true" id="Staphylococcus epidermidis">
<listOfParameters>
<parameter value="-1000" id="cobra_default_lb" sboTerm="SBO:0000626"
constant="true" units="mmol_per_gDW_per_hr"/>
<parameter value="1000" id="cobra_default_ub" sboTerm="SBO:0000626"
constant="true" units="mmol_per_gDW_per_hr"/>
<parameter value="0" id="cobra_0_bound" sboTerm="SBO:0000626"
constant="true" units="mmol_per_gDW_per_hr"/>
</listOfParameters>
```

- Changing a line for each irreversible reaction to declare lower and upper bound parameters as mentioned before.

```
<reaction id="reac_221" name="DIHYDLIPACETRANS-RXN" reversible="false">
```

changes to:

```
<reaction id="reac_221" name="DIHYDLIPACETRANS-RXN" reversible="false"
fbc:upperFluxBound="cobra_default_ub"
fbc:lowerFluxBound="cobra_0_bound">
```

- Propagation of the modification. This can be easily done using the 'sed' command:

```
sed -i 's/reversible="false"/reversible=
"false" fbc:upperFluxBound="cobra_default_ub" fbc:lowerFluxBound=
"cobra_0_bound"/g' S.epidermidis_propagacion-cambio.sbml
```

## 2 Comparisson with other BIGG Models

| Model Name | Specie | Reactions | Metabolites | Blocked Metabolites | Blocked metabolites Ratio (%) |
|---|---|---|---|---|---|
| GSMMM_SupIII | *S.epidermidis* RP62A | 990 | 938 | 277 | 29,53 |
| iYO844 | *B.subtilis subsp. subtilis* str. 168 | 1250 | 990 | 593 | 59,90 |
| iSB619 | *S.aureus subsp. aureus* N315 | 743 | 655 | 293 | 44,73 |
| iNJ661 | *M.tuberculosis* H37Rv | 1025 | 825 | 285 | 34,55 |
| iNF517 | *L.lactis subsp. cremoris* MG1363 | 754 | 650 | 241 | 37,08 |
| iND750 | *S.cerevisiae* S288C | 1266 | 1059 | 633 | 59,77 |
| iMM904 | *S.cerevisiae* S288C | 1577 | 1226 | 689 | 56,20 |
| iML1515 | *E.coli str. K-12* substr. MG1655 | 2712 | 1877 | 971 | 51,73 |
| iEK1008 | *M.tuberculosis* H37Rv | 1226 | 998 | 333 | 33,37 |

Table 0.1: Comparison of blocked metabolites ratio with similar-sized models from BIGG database.

## 3 Other optimization algorithms

**Mixed-Integer Linear Programs (MILP)**

While performing the structural analysis of the network, sometimes it is useful to pose optimization problems in which some variables are forced to have integer values. This kind of problems are called mixed-integer linear programs (MILP). In this approach, the same FBA problems are formulated using MILP instead of LP methods. MILP techniques are less efficient than their LP counterparts and there appear parameters that have to be specified in order to control its behaviour. It is worth noting that setting different values to these parameters can lead to different solutions (even incorrect ones).

These remarks are also true for other optimization methods. So it is desirable to use LP methods when available. However, there are some widely used algorithms that rely on MILP formulations [1].

**Introduction of a biomass lumped reaction.**

Biologically, biomass composition is not always constant; rather they can vary depending on the metabolic state. Similarly, biomass to biofilm ratio can change. Introducing individual biomass components allows us to perform a simulation where biomass composition can vary to obtain an optimal state. These metabolites are internal, in order to be considered in steady-state a transport pseudo-reaction is attached to them. That is, if $M_B = \{m_{i_1}, \cdots, m_{i_k}\}$ are the biomass components, for each $m_{i_j}$ an associated transport pseudo-reaction is introduces

$$r_{i_j} : m_{i_j} \rightarrow$$

When the biomass composition is considered fixed, this analysis can be simplified by introducing a biomass pseudo-reaction to encompass the flux of all those pseudo-reactions:

$$r_B : m_{i_1} + \cdots + m_{i_k} \rightarrow \quad (1)$$

where this reaction stoichiometry reflects the biomass composition.

After including this biomass reaction, the constraints $r_i = c_i \forall r_i \in R_B$ can be replaced by $r_B = 1$.

## 4 Fluctuation of the components uptake of the culture medium for a metabolic state of minimization

### Variations on concentration values

After fixing the minimal total flux as an additional constraint, the possible oscillations of the concentration values of the culture medium's component under minimal total flux condition is summarized in Table 0.1.

| Reaction | minimum uptake | maximum uptake | maximum in Min |
|---|---|---|---|
| AMMONIUM_mm_tx | 0.0 | 0.21 | 0.21 |
| ARG_AA_mm_tx | 0.0 | 0.57 | 0.57 |
| ASN_AA_mm | 0.0 | 1.13 | 1.13 |
| CYS_AA_mm | 0.0 | 0.41 | 0.41 |
| GLC_mm_tx | 0.0 | 11.1 | 11.1 |
| GLT_AA_mm_tx | 0.0 | 01.02 | 01.02 |
| HIS_AA_mm_tx | 0.0 | 0.64 | 0.64 |
| ILE_AA_mm_tx | 0.0 | 1.14 | 0.269 |
| L-ALPHA-ALANINE_AA_mm_tx | 0.0 | 1.12 | 1.12 |
| L-ASPARTATE_AA_mm_tx | 0.0 | 1.13 | 1.13 |
| LEU_AA_mm_tx | 0.0 | 1.14 | 0.282 |
| LYS_AA_mm_tx | 0.0 | 0.68 | 0.336999 |
| MET_AA_mm_tx | 0.0 | 0.67 | 0.091440 |
| NIACINE_mm_tx | 0.0 | 0.16 | 0.001742 |
| PHE_AA_mm_tx | 0.0 | 0.6 | 0.137 |
| PRO_AA_mm_tx | 0.0 | 1.3 | 1.3 |
| SER_AA_mm_tx | 0.0 | 0.95 | 0.95 |
| THR_AA_mm_tx | 0.0 | 1.26 | 1.26 |
| TRP_AA_mm_tx | 0.0 | 0.49 | 0.49 |
| TYR_AA_mm_tx | 0.0 | 0.55 | 0.119 |
| VAL_AA_mm_tx | 0.0 | 1.28 | 1.28 |

In general these variations are as expected: they range between 0 and a value that corresponds to the maximum values for the corresponding exchange reactions. The exception is found in a few amino acids (isoleucine, leucine, lysine, methionine, tyrosine and phenylalanine), and in niacin (vitamin B3), with large differences between the minimum and maximum values that are incorporated into the metabolic network and those obtained in our analysis. Isoleucine, leucine, tyrosine, and phenylalanine are halved, lysine is doubled, methionine is increased by one order of magnitude, and niacin is increased by two orders of magnitude

Moreover, when the oscillations of the input variables are calculated in non-growth conditions ($ATPase = m_{ATP}$), the same fluctuations in nutrient demand are maintained and the same result is obtained when the oscillations of the input flow of the components of the culture medium are calculated without limiting the incorporation of nutrients into the metabolic network.

Table 0.2: Fluctuation of the components uptake of the culture medium for a metabolic state of minimization. First two columns are lower and upper bounds of concentration uptake $gDCW^{-1}$, respectively. The third column is the maximum concentration value uptake $gDCW^{-1}$ for an objective function of minimization.

## 5 Essential reactions

**Essential reactions:**

1. RXN-11065
2. RXN-11291
3. RXN-11295
4. RXN-11296
5. RXN-11297
6. RXN-11339
7. 2-DEHYDROPANTOATE-REDUCT-RXN
8. RXN-12002
9. 2.3.1.157-RXN
10. 2.4.1.53-RXN
11. 2.5.1.19-RXN
12. 2.5.1.64-RXN
13. 2.7.7.39-RXN
14. 2.7.7.40-RXN
15. 3-CH3-2-OXOBUTANOATE-OH-CH3-XFER-RXN
16. 3-DEHYDROQUINATE-DEHYDRATASE-RXN
17. 3-DEHYDROQUINATE-SYNTHASE-RXN
18. 5.4.2.10-RXN_i
19. 6.1.1.13-RXN
20. 6.3.2.10-RXN
21. 6.3.2.7-RXN
22. RXN-15117
23. ADENOSYLHOMOCYSTEINE-NUCLEOSIDASE-RXN
24. RXN-16648
25. RXN-18006
26. ADOMET-DMK-METHYLTRANSFER-RXN

27. RXN-18007
28. AICARSYN-RXN
29. AICARTRANSFORM-RXN
30. AIRS-RXN
31. RXN-18008
32. RXN-18013
33. RXN-18020
34. RXN-18027
35. RXN-18035
36. RXN-18036
37. RXN-18037
38. RXN-18039
39. ASPCARBTRANS-RXN
40. ASPDECARBOX-RXN
41. ATPASE-RXN
42. RXN-8975
43. RXN-8976
44. RXN-8992
45. RXN-8999
46. RXN-9310
47. RXN-9311
48. CARDIOLIPSYN-RXN
49. CDPDIGLYSYN-RXN
50. CHORISMATE-SYNTHASE-RXN
51. RXN66-532_i
52. S-ADENMETSYN-RXN
53. SAICARSYN-RXN
54. SHIKIMATE-5-DEHYDROGENASE-RXN

55. SHIKIMATE-KINASE-RXN
56. DAHPSYN-RXN
57. DALADALALIG-RXN
58. DCDPKIN-RXN
59. TEICHOICSYN2-RXN
60. TEICHOICSYN3-RXN
61. THYMIDYLATESYN-RXN
62. DEPHOSPHOCOAKIN-RXN
63. TRANS-RXN-314
64. DIACYLGLYKIN-RXN_rev
65. DIHYDROFOLATEREDUCT-RXN
66. DIHYDROOROT-RXN
67. UDP-NACMUR-ALA-LIG-RXN
68. UDP-NACMURALA-GLU-LIG-RXN
69. UDPGLCNACEPIM-RXN
70. DIOHBUTANONEPSYN-RXN
71. DIPHOSPHOMEVALONTE-DECARBOXYLASE-RXN
72. UDPNACETYLGLUCOSAMENOLPYRTRANS-RXN
73. UDPNACETYLMURAMATEDEHYDROG-RXN
74. DMK-RXN
75. DTDPKIN-RXN
76. DTMPKI-RXN
77. UNDECAPRENYL-DIPHOSPHATASE-RXN
78. FADSYN-RXN
79. FGAMSYN-RXN
80. FPPSYN-RXN
81. GLCNACPTRANS-RXN
82. GLUC1PADENYLTRANS-RXN

83. GLUC1PURIDYLTRANS-RXN
84. GLYCINE–TRNA-LIGASE-RXN
85. GLYCOGENSYN-RXN
86. GLYRIBONUCSYN-RXN
87. GPPSYN-RXN
88. GTP-CYCLOHYDRO-II-RXN
89. GUANYL-KIN-RXN
90. HYDROXYMETHYLGLUTARYL-COA-SYNTHASE-RXN
91. IMP-DEHYDROG-RXN
92. IMPCYCLOHYDROLASE-RXN
93. INORGPYROPHOSPHAT-RXN
94. IPPISOM-RXN
95. ISOCHORSYN-RXN
96. LUMAZINESYN-RXN
97. MEVALONATE-KINASE-RXN
98. NAD-KIN-RXN
99. NAG1P-URIDYLTRANS-RXN
100. NAPHTHOATE-SYN-RXN
101. NICONUCADENYLYLTRAN-RXN
102. O-SUCCINYLBENZOATE-COA-LIG-RXN
103. O-SUCCINYLBENZOATE-COA-SYN-RXN
104. OROPRIBTRANS-RXN
105. OROTPDECARB-RXN
106. P-PANTOCYSDECARB-RXN
107. PANTEPADENYLYLTRAN-RXN
108. PANTOTHENATE-KIN-RXN
109. PA_synth_NADPH
110. PGPPHOSPHA-RXN

111. PHOSPHAGLYPSYN-RXN
112. PHOSPHOMEVALONATE-KINASE-RXN
113. PIA1_synth
114. PIA2_synth
115. PIA3_synth
116. RXN-18014
117. PRPPAMIDOTRANS-RXN
118. PRPPSYN-RXN
119. RXN-18038
120. Palmitate_synth
121. RIBITOL-5-PHOSPHATE-2-DEHYDROGENASE-RXN
122. RIBOFLAVIN-SYN-RXN
123. RIBOFLAVINKIN-RXN
124. RIBOFLAVINSYNDEAM-RXN
125. RIBOFLAVINSYNREDUC-RXN
126. RIBOPHOSPHAT-RXN
127. RIBOSYLHOMOCYSTEINASE-RXN
128. RXN-10015
129. RXN-10017

**Classification of equivalences between essential reactions:**

There is a total of 37 non-equivalent essential reactions with only 19 essential reactions that have no equivalences between them.

Most of the essential reactions are grouped in 8 clusters that have more than 2 or more reactions related to each other by equivalences. In fact, 109 out of the 128 the essential reactions are included in these clusters.

The following list includes only those clusters that has more than one reaction in them:

- Cluster 1
  - 3-CH3-2-OXOBUTANOATE-OH-CH3-XFER-RXN
  - 2-DEHYDROPANTOATE-REDUCT-RXN
  - DEPHOSPHOCOAKIN-RXN
  - P-PANTOCYSDECARB-RXN
  - PANTEPADENYLYLTRAN-RXN
  - PANTOTHENATE-KIN-RXN
  - ASPDECARBOX-RXN
- Cluster 2
  - 2.3.1.157-RXN
  - 5.4.2.10-RXN
  - NAG1P-URIDYLTRANS-RXN
- Cluster 3
  - TEICHOICSYN2-RXN
  - TEICHOICSYN3-RXN
  - GLCNACPTRANS-RXN
  - RXN-18007
  - TRANS-RXN-314
  - 6.1.1.13-RXN
  - RXN-18008
  - 2.4.1.53-RXN
  - RIBITOL-5-PHOSPHATE-2-DEHYDROGENASE-RXN
  - UDPGLCNACEPIM-RXN
  - RXN-18006
  - 2.7.7.39-RXN
  - 2.7.7.40-RXN
  - RXN-18020
  - RXN-18027

- Cluster 4
  - 3-DEHYDROQUINATE-DEHYDRATASE-RXN
  - 3-DEHYDROQUINATE-SYNTHASE-RXN
  - SHIKIMATE-KINASE-RXN
  - 2.5.1.19-RXN
  - DAHPSYN-RXN
  - CHORISMATE-SYNTHASE-RXN
- Cluster 5
  - DMK-RXN
  - RXN-8992
  - RXN-9310
  - RXN-9311
  - O-SUCCINYLBENZOATE-COA-LIG-RXN
  - ISOCHORSYN-RXN
  - O-SUCCINYLBENZOATE-COA-SYN-RXN
  - S-ADENMETSYN-RXN
  - RIBOSYLHOMOCYSTEINASE-RXN
  - ADENOSYLHOMOCYSTEINE-NUCLEOSIDASE-RXN
  - 2.5.1.64-RXN
  - ADOMET-DMK-METHYLTRANSFER-RXN
  - NAPHTHOATE-SYN-RXN
  - RXN-10015
  - RXN-10017
- Cluster 6
  - RXN-11295
  - RXN-11296
  - RXN-11297
  - RXN-11339
  - UDPNACETYLMURAMATEDEHYDROG-RXN
  - GLYCINE–TRNA-LIGASE-RXN
  - 6.3.2.10-RXN
  - 6.3.2.7-RXN
  - UDP-NACMUR-ALA-LIG-RXN
  - UDP-NACMURALA-GLU-LIG-RXN

  - DALADALALIG-RXN
  - UDPNACETYLGLUCOSAMENOLPYRTRANS-RXN
  - RXN-8975
  - RXN-8976
  - UNDECAPRENYL-DIPHOSPHATASE-RXN
- Cluster 7
  - AIRS-RXN
  - SAICARSYN-RXN
  - FGAMSYN-RXN
  - GLYRIBONUCSYN-RXN
  - PRPPAMIDOTRANS-RXN
  - AICARSYN-RXN
- Cluster 8
  - IMPCYCLOHYDROLASE-RXN
  - AICARTRANSFORM-RXN
- Cluster 9
  - OROTPDECARB-RXN
  - OROPRIBTRANS-RXN
  - ASPCARBTRANS-RXN
  - DIHYDROOROT-RXN
- Cluster 10
  - PHOSPHAGLYPSYN-RXN
  - CDPDIGLYSYN-RXN
  - PGPPHOSPHA-RXN
- Cluster 11
  - DTDPKIN-RXN
  - DTMPKI-RXN
  - DIHYDROFOLATEREDUCT-RXN
  - THYMIDYLATESYN-RXN
- Cluster 12
  - FADSYN-RXN
  - DIOHBUTANONEPSYN-RXN

  - RIBOPHOSPHAT-RXN
  - RIBOFLAVIN-SYN-RXN
  - RIBOFLAVINKIN-RXN
  - RIBOFLAVINSYNREDUC-RXN
  - LUMAZINESYN-RXN
  - GTP-CYCLOHYDRO-II-RXN

- Cluster 13
  - IPPISOM-RXN
  - PHOSPHOMEVALONATE-KINASE-RXN
  - MEVALONATE-KINASE-RXN
  - FPPSYN-RXN
  - GPPSYN-RXN
  - DIPHOSPHOMEVALONTE-DECARBOXYLASE-RXN

- Cluster 14
  - GLUC1PADENYLTRANS-RXN
  - GLYCOGENSYN-RXN

- Cluster 15
  - Palmitate_synth
  - PA_synth_NADPH

- Cluster 16
  - RXN-8999
  - RXN-11065
  - RXN-11291

- Cluster 17
  - RXN-18013
  - RXN-18014

- Cluster 18
  - RXN-18037
  - RXN-18037
  - RXN-18039
  - RXN-18035
  - RXN-18036

Primary implications

- PIA2_synth
- NAD-KIN-RXN
- DIACYLGLYKIN-RXN
- PIA3_synth
- CARDIOLIPSYN-RXN
- DCDPKIN-RXN
- NICONUCADENYLYLTRAN-RXN
- ATPASE-RXN
- 3-CH3-2-OXOBUTANOATE-OH-CH3-XFER-RXN
- TEICHOICSYN2-RXN
- DMK-RXN
- DTDPKIN-RXN
- FADSYN-RXN
- GLUC1PADENYLTRANS-RXN
- RXN-8999
- RXN-18037

Between these indirect implications we can distinguish between secondary implications (those which imply the primary ones), tertiary (which imply the secondary ones), etc. Looking at these levels of implications with respect to biomass precursors, it can be observed that there are 12 primary implications, numerous secondary ones, and a few tertiary and only one quaternary. In addition, there is a primary implication at the level of the INORGPYROPHOSPHAT-RXN reaction that participates in numerous secondary and tertiary implications. All this information is sumarized in the Supplementary materials.

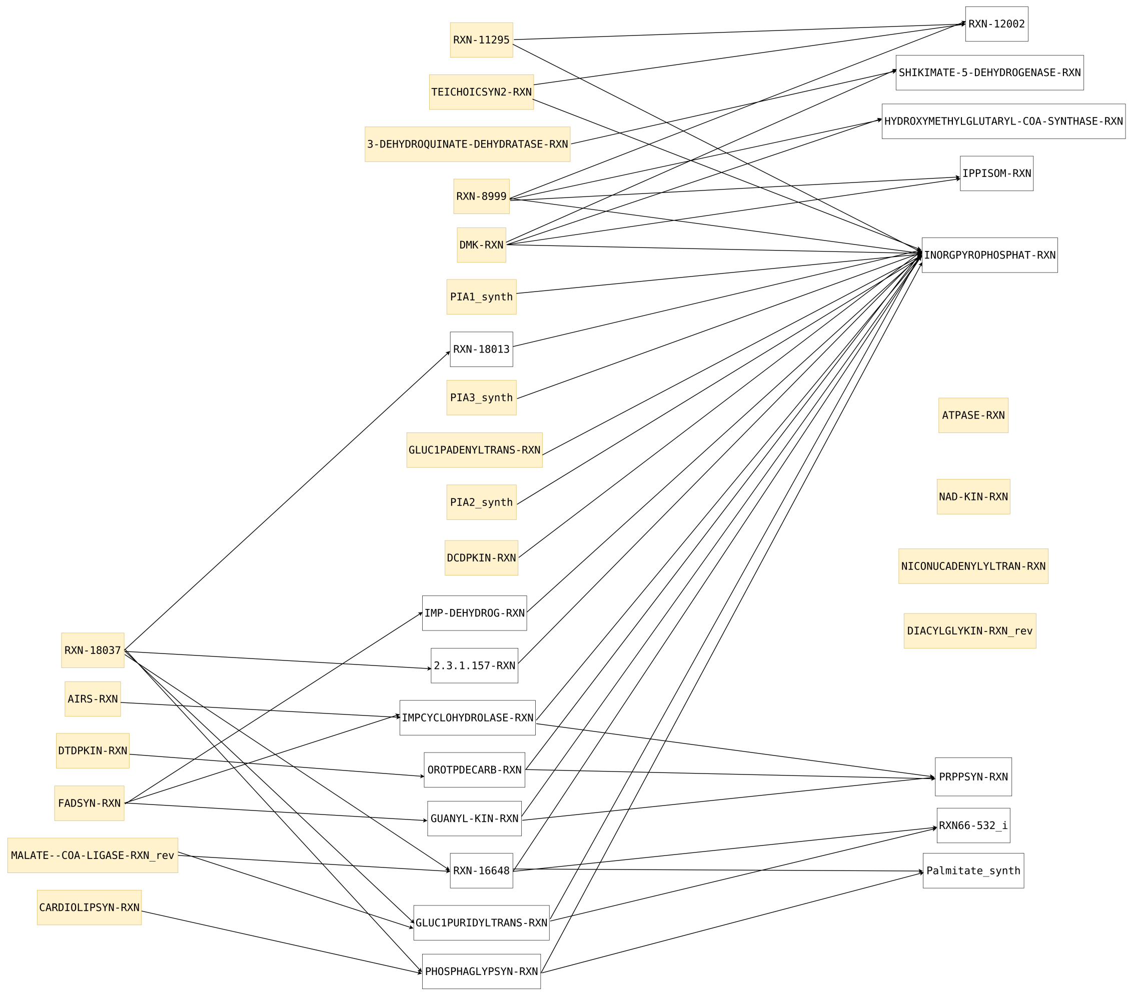


Figure 0.1: Biomass implications.

From right to left: primary implications, secondary implications, tertiary implications. Yellow-colored boxes are those implications that do not participate in further implications.

## 6 The cysteine and sulfate cut set

Due to the importance of these two reactions, we have also studied which of the essential reactions required the sulfate ion (RIB5PISOM-RXN) and which cysteine (8.4.8-RXN_i, 325-BISPHOSPHATE-NUCLEOTIDASE-RXN, ADENYLYLSULFKIN-RXN, SULFATE-ADENYLYLTRANS-RXN, SULFITE-REDUCT-RXN; All belong to the cysteine biosynthetic pathway).

Another interesting aspect was to check which biomass reactions were blocked in the absence of cysteine and sulfate. These reactions were as follows: CYS_AA_bm_tx, CoA_bm_tx, MET_AA_bm_tx, acCoA_bm_tx and succCoA_bm_tx. Implications with sulfate and cysteine were also observed. 7 implications were obtained: 2-DEHYDROPANTOATE-REDUCT-RXN, 3-CH3-2-OXOBUTANOATE-OH-CH3-XFER-RXN, ASPDECARBOX-RXN, DEPHOSPHOCOAKIN-RXN, P-PANTOCYSDECARB-RXN, PANTEPADENYLYLTRAN-RXN and PANTOTHENATE-KIN - RXN.

## 7 Hybrid cut sets

- SULFATE_mm_tx,RIB5PISOM-RXN
- ASN_AA_mm_tx,RXN490-3616
- ASN_AA_mm_tx,6.3.5.6-RXN
- ASN_AA_mm_tx,RXN-12460
- CYS_AA_mm_tx,1.8.4.8-RXN
- CYS_AA_mm_tx,325-BISPHOSPHATE-NUCLEOTIDASE-RXN
- CYS_AA_mm_tx,ADENYLYLSULFKIN-RXN
- CYS_AA_mm_tx,SULFATE-ADENYLYLTRANS-RXN
- CYS_AA_mm_tx,SULFITE-REDUCT-RXN
- HIS_AA_mm_tx,HISTAMINOTRANS-RXN
- HIS_AA_mm_tx,HISTCYCLOHYD-RXN
- HIS_AA_mm_tx,HISTIDPHOS-RXN
- HIS_AA_mm_tx,HISTPRATPHYD-RXN
- HIS_AA_mm_tx,PRIBFAICARPISOM-RXN
- HIS_AA_mm_tx,GLUTAMIDOTRANS-RXN
- HIS_AA_mm_tx,IMIDPHOSDEHYD-RXN
- HIS_AA_mm_tx,ATPPHOSPHORIBOSYLTRANS-RXN
- ILE_AA_mm_tx,ACETOOHBUTREDUCTOISOM-RXN
- ILE_AA_mm_tx,ACETOOHBUTSYN-RXN

- ILE_AA_mm_tx,DIHYDROXYMETVALDEHYDRAT-RXN
- ILE_AA_mm_tx,BRANCHED-CHAINAMINOTRANSFERILEU-RXN
- LEU_AA_mm_tx,BRANCHED-CHAINAMINOTRANSFERLEU-RXN
- LEU_AA_mm_tx,2-ISOPROPYLMALATESYN-RXN
- LYS_AA_mm_tx,TETHYDPICSUCC-RXN
- LYS_AA_mm_tx,RXN-14014
- LYS_AA_mm_tx,DIAMINOPIMDECARB-RXN
- LYS_AA_mm_tx,DIAMINOPIMEPIM-RXN
- LYS_AA_mm_tx,SUCCDIAMINOPIMDESUCC-RXN
- LYS_AA_mm_tx,DIHYDRODIPICSYN-RXN
- LYS_AA_mm_tx,SUCCINYLDIAMINOPIMTRANS-RXN
- LYS_AA_mm_tx,ASPARTATE-SEMIALDEHYDE-DEHYDROGENASE-RXN
- LYS_AA_mm_tx,ASPARTATEKIN-RXN
- MET_AA_mm_tx,RXN-5061
- MET_AA_mm_tx,HOMOCYSMETB12-RXN
- MET_AA_mm_tx,HOMOSERINE-O-ACETYLTRANSFERASE-RXN
- MET_AA_mm_tx,ASPARTATE-SEMIALDEHYDE-DEHYDROGENASE-RXN
- MET_AA_mm_tx,ASPARTATEKIN-RXN
- NIACINE_mm_tx,OXYGEN-MOLECULE_tx
- NIACINE_mm_tx,L-ASPARTATE-OXID-RXN
- NIACINE_mm_tx,QUINOPRIBOTRANS-RXN
- NIACINE_mm_tx,QUINOLINATE-SYNTHA-RXN
- PHE_AA_mm_tx,PREPHENATEDEHYDRAT-RXN
- PHE_AA_mm_tx,CHORISMATEMUT-RXN
- TYR_AA_mm_tx,PREPHENATEDEHYDROG-RXN
- TYR_AA_mm_tx,TYROSINE-AMINOTRANSFERASE-RXN
- TYR_AA_mm_tx,CHORISMATEMUT-RXN
- VAL_AA_mm_tx,ACETOLACTSYN-RXN
- VAL_AA_mm_tx,DIHYDROXYISOVALDEHYDRAT-RXN
- VAL_AA_mm_tx,ACETOLACTREDUCTOISOM-RXN
- CYS_AA_mm_tx,ADPREDUCT-RXN,THIOREDOXIN-REDUCT-NADPH-RXN
